# Association Analysis of 14 Candidate Gene Polymorphism with Depression and Stress among Gestational Diabetes Mellitus

**DOI:** 10.3390/genes10120988

**Published:** 2019-11-30

**Authors:** Kai Wei Lee, Siew Mooi Ching, Vasudevan Ramachandran, Maiza Tusimin, Noraihan Mohd Nordin, Seng Choi Chong, Fan Kee Hoo

**Affiliations:** 1 Department of Family Medicine, Faculty of Medicine and Health Sciences, Universiti Putra Malaysia, 43400 Serdang, Selangor, Malaysia; lee_kai_wei@yahoo.com; 2 Malaysian Research Institute on Ageing, Universiti Putra Malaysia, 43400 Serdang, Selangor, Malaysia; vasudevan@upm.edu.my; 3 Department of Obstetrics and Gynaecology, Faculty of Medicine and Health Sciences, Universiti Putra Malaysia, 43400 Serdang, Selangor, Malaysia; maiza@upm.edu.my; 4 Department of Obstetrics and Gynaecology, Hospital Kuala Lumpur, 50586 Kuala Lumpur, Malaysia; dr.noraihan@moh.gov.my; 5 Department of Psychiatry, Faculty of Medicine and Health Sciences, Universiti Putra Malaysia, 43400 Serdang, Selangor, Malaysia; sengchoi@upm.edu.my; 6 Department of Medicine, Faculty of Medicine and Health Sciences, Universiti Putra Malaysia, 43400 Serdang, Selangor, Malaysia; fan_kee@upm.edu.my

**Keywords:** polymorphisms, genetic variation, depression, anxiety, stress, gestational diabetes

## Abstract

The association of candidate genes and psychological symptoms of depression, anxiety, and stress among women with gestational diabetes mellitus (GDM) in Malaysia was determined in this study, followed by the determination of their odds of getting psychological symptoms, adjusted for socio-demographical background, maternal, and clinical characteristics. Single nucleotide polymorphisms (SNPs) recorded a significant association between SNP of *EPHX2* (rs17466684) and depression symptoms (AOR = 7.854, 95% CI = 1.330–46.360) and stress symptoms (AOR = 7.664, 95% CI = 1.579–37.197). Associations were also observed between stress symptoms and SNP of *OXTR* (rs53576) and (AOR = 2.981, 95% CI = 1.058–8.402) and SNP of *NRG1* (rs2919375) (AOR = 9.894, 95% CI = 1.159–84.427). The SNP of *EPHX2* (rs17466684) gene polymorphism is associated with depression symptoms among Malaysian women with GDM. SNP of *EPHX2* (rs17466684), *OXTR* (rs53576) and *NRG1* (rs2919375) are also associated with stress symptoms.

## 1. Introduction

Gestational diabetes mellitus (GDM) is one of the common complications in pregnancy. Its prevalence in Asia is 11.5% [1]. GDM is a known risk factor for neonatal adverse outcomes [2,3,4]. Additionally, a diagnosis of GDM is a stressful life event [5,6,7,8] which has an adverse impact on self-perception towards health and quality of life [6,9]; as well as increased odds of experiencing emotional distress. Previous studies reported that the prevalence of depression among women sufferers from GDM stood at 56.7%, while anxiety was 57.7%, and stress was even higher at 62.8% [10,11,12]. GDM and perinatal mental problems undeniably affect all members of the family [13]. This mental condition may reoccur or worsen to postpartum depression [14]. Multiple determinants such as socio-demographical background, maternal and clinical profiles have a reported positive association with psychological symptoms [15,16,17,18,19].

Genetic factors clearly play a substantial role in the etiology of psychological symptoms of depression, anxiety and/or stress, as evidenced by other studies, which indicate a heritability ranges from 45% to 50% for these disorders [20,21,22]. The genetic profile of the mother is particularly important if she wants to determine whether her child will be predispose to psychological disorders in the future. However, it is challenging to identify particular genetic variants underlying for symptoms of depression, anxiety and/or stress susceptibility because their psychological symptoms are not caused by single gene, but a complex interaction among multiple genes, socio-demographic background, clinical, and biological moderators [23]. The candidate gene-by-environment interaction hypothesis regarding psychological symptoms of depression, anxiety and/or stress has received widespread attention and acclaim; therefore, many studies to date have used this approach to underpin their findings for genetic effects on psychological symptoms of depression, anxiety, and/or stress [24].

Indeed, it is not difficult to find studies which have reported a significant association between candidate genes and these psychological symptoms, such as brain-derived neurotrophic factor (*BDNF*) [25,26] and oxytocin receptor genes (*OXTR*) [27,28]. These genes may be associated with depression or anxiety; however, there are ample studies which have failed to replicate the same results in the candidate gene literature [29,30,31]. One explanation for this lack of success in producing the replicable main effect of these genes is that the certain genetic variants are highly dependent on the gender, population, and disease-related outcomes [32]; even though these studies have recruited patients with major depressive disorder [27,28,29,30,31,32,33,34,35,36,37,38,39,40,41]; anxiety disorder [42,43,44]; and post-traumatic stress disorders [45] diagnosed according to Diagnostic and Statistical Manual of Mental Disorders and/or International Statistical Classification of Diseases. This has led to increasing skepticism about the true association or lack thereof between candidate genes and psychological symptoms of depression, anxiety and/or stress. Without testing the candidate genes in our population, it is difficult to conclude that the previous results are also applicable in our samples. One strategy that may aid in identifying the candidate genes in association with symptoms of depression, anxiety and/or stress is to interrogate several candidate genes thought to be associated with the underlying psychological symptoms of depression, anxiety and/or stress. To this end, we have constructed a custom of SNP array containing 18 genes that were chosen based on hypotheses regarding biological systems of relevance to depression [46,47,48,49,50]; anxiety [42,51,52] and stress [45,53]. These custom SNPs provide excellent coverage of many previously suggested and functionally important candidate genes for depression, anxiety and stress, including *NPY5R* [42,52]; *ANO2* [42]; *EPHX2* [42,51]; *TPH2* [35]; *NRG1* [34]; *LHPP* [38,39,54]; *FKBP5* [41,45]; *SDK2* [42]; *RORA* [33,55]; *OXTR* [27,28]; *BDNF* [56,57]; *HTR2C* [43]; *TEX51* [42]; and *PLEKHG1* [42]. Many of the genes represented on the array have also been reported to be involved in associated heritable phenotypes that are associated with symptoms of depression, anxiety and/or stress. Despite that, the putative susceptibility genes for depression, anxiety or stress have yet to be definitively identified among GDM women.

In light of the complications caused by GDM itself and the devastating consequences of depression and related psychological symptoms of anxiety and stress among women with GDM, we suggest performing a study of fourteen candidate genes to elucidate its genotypic effect on symptoms of depression, anxiety and/or stress among GDM women. The aim of the present study was to perform candidate gene analysis via mass array to evaluate the associations, if any, between phenotypes of threes psychological symptoms and fourteen candidate genes, as adjusted for socio-demographical background, maternal and clinical profile among GDM women.

## 2. Materials and Methods

### 2.1. Study Population

We performed a post-hoc exploratory sub-analysis of a cross-sectional study among GDM women (n = 343) to check which candidate SNPs may be associated with symptoms of depression, anxiety and/or stress in this particular population. We conducted a genetic association study using the cross-sectional study from the previously described “Prevalence and factors associated with depressive, anxiety and stress symptoms among women with gestational diabetes mellitus in tertiary care centres: A cross-sectional study”, which was conducted between July 2018 and October 2018 in Malaysia [58]. The study participants were women enrolled in second or third trimester care and diagnosed with GDM at Hospital Kuala Lumpur or Hospital Serdang. All participants were native Malaysians and residents of surrounding areas. The detailed study protocol has been described previously [58]. In that study, 526 women agreed to participate. Upon completion of sample collection and analysis, data for depression, anxiety and stress score and polymorphisms of candidate genes were available for a total of 343 participants.

The general inclusion criteria were that the pregnant women were Malaysian, aged ≥18 years old, with a diagnosis of GDM. The diagnosis of GDM is defined as fasting plasma glucose ≥5.1 mmol/L or 75 g two-hours oral glucose tolerance test ≥ 7.8 mmol/L according to Malaysian Clinical Practice Guidelines [59,60]. The exclusion criteria were those with pre-existing diabetes.

Regarding patients and controls, patients with depression were defined as those with the DASS depression subscale score ≥10; otherwise, they were in control group if scoring <10 in the DASS-depression subscale. Similarly, they were categorized as a patient for anxiety if they scored ≥8 in the DASS anxiety subscale; they were in control group if the score was <8. They were categorized as a patient for stress if they scored ≥15 in the DASS stress subscale, and placed in the control group if scoring <15 in the DASS stress subscale.

### 2.2. Socio-Demographic Background and Clinical Characteristics

Socio-demographic backgrounds and clinical characteristics were recorded at enrollment to obtain information related to maternal profile, past-obstetrics history, concurrent medical problems, and family history. These data were obtained from the self-administered questionnaire and medical records.

### 2.3. Measurement of Depression, Anxiety and Stress Symptoms

The detailed sampling and assessment of depression, anxiety, and stress symptoms have been previously described [58]. We used an English [61] and Malay [62] version of the validated questionnaire on Depression, Anxiety, and Stress-21 items (DASS-21). DASS-21 is a valid and reliable measure to screen for depression, anxiety, and stress symptoms among both non-clinical and clinical populations. The English version of the questionnaire (DASS-21) has strong validation, with Cronbach’s alpha values of 0.72 for depression; 0.77 for anxiety; and 0.70 for stress, and the overall Cronbach’s alpha for DASS-21 is 0.88 [61]. The translated Malay version of the DASS-21 questionnaire has good Cronbach’s alpha values, as well as among the Malaysian population (0.84 for depression; 0.74 for anxiety; and 0.79 for stress) [62] and among diabetic patients (0.75 for depression; 0.74 for anxiety; and 0.79 for stress) [63]. The participants rated on a 4-point severity scale their experiences over the preceding week. Scores for subscale for depression, anxiety, and stress were calculated. The depression symptoms defined to follow the depression subscale, ≥10; anxiety symptoms, ≥8; and stress symptoms, ≥15 [61].

### 2.4. Blood Sample Collection and DNA Extraction

Samples of 5 mL of blood were collected from the participants’ peripheral blood using a 21-gauge needle with a 5.0 mL syringe by a qualified phlebotomist into EDTA tubes (Becton Dickinson, East Rutherford, NJ, USA). The samples were kept in portable icebox at 4 °C during the transportation and there were stored at −20 °C in laboratory for further analysis. Genomic DNA was isolated by using the QIAamp Blood DNA Mini Kit (QIAGEN, Hilden, Germany). The quantity and purity of extracted DNA were checked using a Biophotometer (Eppendorf, Hamburg, Germany). First, readings of a blank using distilled water against A260 and A280 of the genomic DNA were obtained. The DNA absorbed UV light with a maximum absorbance of 260 nm, while the protein absorbed UV light with a maximum absorbance of 280 nm. By dividing the amount of UV absorption at 260 nm by the absorption at 280 nm, the standard measure of the purity of the genomic DNA could be calculated. The genomic DNA was measured to be relatively free of protein impurity when the ratio of optical density was between 1.7 and 2.0.

### 2.5. Mass Array Genotyping

Genes candidates were selected based on previous data implicating an association with the studies SNPs and clinical syndrome of depression [27,28,29,30,31,32,33,34,35,36,37,38,39,40,41]; anxiety [42,43,44] or stress [45] diagnosed according to Diagnostic and Statistical Manual of Mental Disorders and/or International Statistical Classification of Diseases. The genotyping analysis of candidate genes polymorphism was analyzed using Agene^®^ MassARRAY platform. SNP analysis was analyzed by Typer Analyzer. Details of candidate genes (location and sequence of SNP) were shown in Table A1.

### 2.6. Statistical Analysis

We used IBM SPSS Statistics version 21.0 to perform the data analysis. A chi-square goodness-of-fit test was performed to assess the agreement of the genotype distribution among candidate genes using Hardy–Weinberg equilibrium, if the *p-*value for chi-square goodness-of-fit tests is significant (*p* < 0.05), the population is not in Hardy–Weinberg equilibrium. If the genotype distribution of candidate genes is not fit to Hardy–Weinberg equilibrium based on equal distribution, expected values for genotype distribution will be adjusted according to the global population. Univariate analysis was used to analyze the association between candidate genes and the presence of depression, anxiety, or stress symptoms among GDM women. The significant difference was set to *p*-value < 0.05. In addition, we tested the candidate gene polymorphism associations with depression phenotypes and any polymorphism adjusted for socio-demographical and clinical moderator effects. Variables with a *p*-value of less than 0.25 in univariate analysis underwent multiple logistic regression [64], because a *p*-value set at <0.05 may miss any variables known to be important [65,66]. A backward stepwise regression method was used [67]. All analyses were made with a 95% CI, and the level of significance was set at *p*  < 0.05.

### 2.7. Ethical Consideration

The study was conducted after written informed consent was obtained from all participants. The Medical Research Ethics Committee (MREC), Ministry of Health Malaysia approved the study protocol (NMRR-17-2264-37814).

## 3. Results

Overall, we found that almost 50% of women with GDM suffered from anxiety symptoms, which was notably higher than symptoms of either depression (13.4%) or stress (11.7%). We also found a significant association between a specific SNP of gene *EPHX2* and depression, as well as SNPs of *EPHX2*, *OXTR*, *NRG1* with stress symptoms.

Analyses of the socio-demographic background and clinical characteristics of the final 343 participants were stratified by psychological problem, as shown in Table 1. Among the various backgrounds and clinical characteristics evaluated, significant differences were observed only in terms of self-monitoring with a glucometer, ethnicity, religion, marital status, underlying with allergy and family history of depression and anxiety (*p* < 0.05) in between those with and without depression symptoms. After a Bonferroni adjustment in the context of family-wise error, these variables (ethnicity, religion, marital status, underlying with allergy and family history of depression and anxiety) still had an adjusted *p*-value < 0.05, except self-monitoring with glucometer (*p*-value = 0.08). Likewise, there were significant differences among ethnicity, religion, smoking habit, and underlying asthma among those with and without anxiety symptoms (*p* < 0.05). After a Bonferroni adjustment in the context of family-wise error for anxiety symptoms among GDM women, variables with adjusted *p*-value < 0.05 included ethnicity and smoking habit, while adjust *p*-value for religion was 0.066 and underlying asthma (*p*-value = 0.058). Further, significant differences were observed in terms of religion, past history of GDM and underlying allergy among those with and without stress symptoms (*p* < 0.05). After a Bonferroni adjustment in the context of family-wise error for stress symptoms among GDM women, the adjusted *p*-value for religion was 0.073, with past history of GDM (*p*-value = 0.048) and underlying allergy history (*p*-value < 0.0001). Bonferroni correction was used to reduce risk of multiple testing error. Even though some of the variables (self-monitoring with glucometer in depression, religion, and underlying asthma in anxiety symptoms) showed significant results with *p*-values < 0.05 after Bonferroni correction, we still proceeded with multiple logistic regression as we did not want to miss any variables known to be important as one of the predictors in our study.

The distribution of candidate gene genotypes satisfied the Hardy–Weinberg equilibrium (*p* > 0.05) (Table A2). Analyses of the genotypes in SNPs of genes *EPHX2, NPY5R, ANO2, NRG1, FKBP5, RORA, OXTR* and *BDNF* among women with GDM were stratified by psychological symptoms and for candidate genotypes with *p*-value > 0.25 using univariate analysis is shown in Table 2. The analyses of the genotypes in SNPs of genes *LHPP, SDK2, HTR2C, TEX51, PLEKHG1* and *TPH2* genotype among women with GDM stratified by presence of psychological symptoms with *p*-value > 0.25 using univariate analysis are shown in (Table A3).

Notably, the proportion of the TT or TC genotypes was higher than that of the CC genotype in SNP of *NRG1* (T > C in rs17466684) among GDM women with stress symptoms (13.2% versus 2.2%; *p* = 0.031). Similarly, the proportion of the TT genotype was higher compared with TG or GG genotypes in the SNP of *FKBP5* (T > G in rs3800373) among GDM women with stress symptoms (57.5% versus 42.5%; *p* = 0.047) as shown in Table 2. On the other hand, there was no significant association between SNPS for candidate genes: [*EPHX2, NPY5R, ANO2, FKBP5* (rs947008), *RORA*, *OXTR* and *BDNF*] and stress symptoms (*p* > 0.05). There was also no association between candidate genes and depression or anxiety symptoms (*p* > 0.05).

The association between specific SNPs’ genotype of candidate genes and psychological symptoms of depression, anxiety and/or stress adjusted for socio-demographical and clinical moderators is shown in Table 3. GDM women with the AA genotype in specific SNP of *EPHX2* (G > A in rs17466684) are 7.9 times more likely to suffer from depression symptoms compared to those who carry G allele in the SNP, when adjusted for ethnicity, religion, practice of home glucose monitoring, planned pregnancy, marital status, past obstetric history of abortion, underlying with allergy, a family history of depression and anxiety and GDM. Likewise, GDM women with the AA genotype in specific SNP of *EPHX2* (G > A in rs17466684) is at 7.7 times odds more likely of getting stress symptoms compared to those who carry G allele in the SNP adjusted for ethnicity, religion, marital status, treatment regimens, past obstetric history of GDM, underlying with allergy and asthma and a family history of depression and anxiety. Not only that, we also found that GDM women with the either AA or AG genotypes in specific SNP of *OXTR* (A > G in rs53576) are 3.0 times more likely to suffer from stress symptoms compared to those who carry GG genotype in the SNP, as well as to those who carry either TT or TC genotypes in SNP of *NRG1* (T > C in rs2919375), is at 9.9 times odds to experience stress symptoms compared to those who carry CC genotype in the SNP.

After a Bonferroni adjustment in the context of family-wise error for depression symptoms among GDM women, the adjusted *p*-value for self-monitoring with glucometer was 0.083, ethnicity (*p*-value = 0.003), religion (*p*-value = 0.004), marital status (*p*-value = 0.012), allergy history (*p*-value = 0.031) and family history of depression and/or anxiety (*p*-value = 0.002).

After a Bonferroni adjustment in the context of family-wise error for anxiety symptoms among GDM women, the adjusted *p*-value for ethnicity with was 0.004, religion (*p*-value = 0.066), smoking habit (*p*-value = 0.007), and asthma (*p*-value = 0.058).

After a Bonferroni adjustment in the context of family-wise error for stress symptoms among GDM women, the adjusted *p*-value for religion was 0.073, history of GDM (*p*-value = 0.048), and allergy (*p*-value < 0.0001).

After a Bonferroni adjustment in the context of family-wise error for stress symptoms among GDM women, the adjusted *p*-value for *NRG1* (rs2919375) was 0.066.

## 4. Discussions

Over the years, an increasing number of polymorphisms in candidate genes related to the psychological problems have been discovered. Even so, most candidate gene association studies have been either overpowered or underpowered to detect the odds of genotypic heterogeneity for psychological symptoms. In this study, we performed simple logistic regression for every candidate gene, followed by multiple logistic regressions to elucidate the actual effect size of genotypes on the presence of depression, anxiety and/or stress symptoms. To our knowledge, this is the first study to examine the symptoms of depression, anxiety and/or stress among GDM women in Malaysia, and is also the first study to use the gene-environmental interaction hypothesis.

It is noteworthy that anxiety symptoms were the most commonly reported symptoms among the population of pregnant women with GDM (57.4% vs. 42.6%), whereas depressive symptoms (86.6% vs. 13.4%) and stress (88.3% vs. 11.7%) were much lower.

Based on logistic regression in this study, we reported that there is significant between SNP (rs17466684) of Epoxide Hydrolase 2 gene (*EPHX2*) with depression symptoms (AOR = 7.854, 95% CI = 1.330–46.360) and stress symptoms (AOR = 7.664, 95% CI = 1.579–37.197). This is different finding compared with a study done in Japan where the carrier of AA genotype in SNP (rs17466684) of *EPHX2* was found to be a risk variant of anxiety particularly panic disorder [42,68]. However, according to our genotypic analysis, this candidate gene was not associated with anxiety symptoms among Malaysian women. Polymorphism in *EPHX2* contributes to the odds of suffering from depression, anxiety, and stress symptoms in the Japanese and Malaysian population. A possible explanation for these findings is that *EPHX2* encodes for a key gat-keeper enzyme (soluble epoxide hydrolase) which functions in the catabolism of epoxy-fatty acids to their corresponding diols [69,70,71]. Soluble epoxide hydrolase is localized in neurons of central amygdala and this enzyme plays a vital role in neuronal firing [72] and it is hence believed that polymorphism in *EPHX2* reduce the potency of anti-inflammatory activity of epoxy-fatty acids in brain [73], thus affecting the release of functional neurotransmitters that influence neuropsychiatric disorders [74].

Neuregulin 1 (*NRG1*) is an important gene signaling numerous neurodevelopment processes such as neurotransmitter receptor expression regulation and synaptic plasticity [75]. In our study, there was a significant association between SNP (rs2919375) of *NRG1* and stress symptoms (AOR = 9.894, 95% CI = 1.159–84.427). To date, the C allele in SNP of *NRG1* (T > C in rs2919375) is a minor allele and also a risk allele for major depression disorder among the Han Chinese population [34] was not found in our study. The reason for this difference is unknown. Apart from the population factor, the possible reason might be due to minor allele frequency in this study was 0.366, compared to 0.410 among Han Chinese population [34], therefore the effect of risk allele or genotype might be underestimated in our study. The minor allele frequency has influent on the power to detect genetic effects, SNPs with minor allele frequency ranges from 25% to 50% might give a false-positive rate ranging from 69.2% to 70.8% [76]. Therefore, the analysis for genes *NRG1* (T > C in rs2919375) indicates that either TT or TC genotypes are determinants for stress symptoms, which might inflate false positive concerns.

Oxytocin receptor genes (*OXTR*) were found to have an association with neuropsychiatry disorders [27,28]; a possible explanation is that *OXTR* regulates the expression of OXTR p53, a potent transcription factor for the oxytocinergic pathway in neurons [77,78,79]. Emerging evidence also shows that *OXTR* rs53576 was associated with the structural coupling of the hypothalamus and amygdala, alteration to this structure is potentially to inflict neuropsychiatric disorders [80,81,82]. In our study, we found a positive association between *OXTR* rs53576 and stress symptoms among GDM women. Our finding contradicts with previous studies among the Japanese population [27] and Caucasian in Italy [28]. In a Japanese study, the G allele is the minor allele and presence of either AA or AG genotypes in SNP rs53576 were associated with panic disorders among the Japanese population [27]. In comparison to the finding done among Caucasians in Italy, a allele is a minor allele among Caucasians in Italy and the presence of either AA or AG genotypes is the protective factor for depression (OR = 0.67, 95% CI = 0.45–0.99) [28].

The findings of this study are of potential clinical and scientific importance as the identification of a significant association between particular candidate genes polymorphism with depression and stress among GDM women in Malaysia have certainly helped in the understanding of genetic aetiology among GDM women in local settings. Future studies should be conducted to validate the value of these candidate genes polymorphism in terms of genetic screening, so that the clinicians can send those GDM women at risk of having depression and stress for a genetic study.

### Study Strength and Limitations

The present study contains multiple logistic regression analysis, adjusted for all socio-demographic backgrounds, and maternal and clinical profiles that potentially modulate the presentation of psychological symptoms. Therefore, the results shown on significant genotype related to depression and stress symptoms are clinically relevant despite this is an unmatched comparative case-control study, a sub-analysis from a cross-sectional study. The study demonstrates an association between candidate genes and the presence of depression, anxiety, or stress symptoms among GDM women. The interpretation of these association is limited by the screening nature of the psychometric tools used in measuring for these psychological symptoms, and not the diagnoses per se. Thus, the results should be interpreted cautiously. Future studies should be conducted with the inclusion of more SNP numbers per candidate gene to confirm the epigenetics-environmental moderator effects.

## 5. Conclusions

A significant association was observed between SNP (rs17466684) of *EPHX2* and depression symptoms when adjusted for ethnicity, religion, the practice of home glucose monitoring, planned pregnancy, marital status, past obstetric history of abortion, underlying with allergy, a family history of depression, and anxiety with GDM. SNPs in *EPHX2* (rs17466684), *OXTR* (rs53576) and *NRG1* (rs2919375) are also associated with stress symptoms adjusted for ethnicity, religion, marital status, treatment regimens, past obstetric history of GDM, underlying with allergy and asthma and a family history of depression and anxiety.

## Figures and Tables

**Table 1:** Univariate analysis on the socio-demographic background and clinical characteristics of the participants with stratification by presence of psychological symptoms (n = 343).

Parameters		Depression			Anxiety			Stress		
		Without Symptoms n = 297 (86.6%)	With Symptoms n = 46 (13.4%)	*p*-Value	Without Symptoms n = 197 (57.4%)	With Symptoms n = 146 (42.6%)	*p*-Value	Without Symptoms n = 303 (88.3%)	With Symptoms n = 40 (11.7%)	*p*-Value
***Treatment Profile***										
Treatments	OAD and/or diet modification	212(87.6)	30(12.4)	0.393	142 (58.7)	100 (41.3)	0.471	217 (89.7)	25 (10.3)	0.234 ^a^
	Insulin with/out OAD and/or diet modification	85(84.2)	16(15.8)		55 (54.5)	46 (45.5)		86 (85.1)	15 (14.9)	
Self-Monitoring with Glucometer	No	46 (80.7)	11(19.3)	0.041 ^a^	33 (57.9)	24 (42.1)	0.841	50 (87.7)	7 (12.3)	0.624
	Yes	198 (90.4)	21 (9.6)		130 (59.4)	89 (40.6)		197 (90.0)	22 (10.0)	
***Socio-Demographic Factors***										
Age		32.17 ± 5.08	31.80 ± 4.65	0.645	32.39 ± 5.04	31.73 ± 4.97	0.259	32.20 ± 5.00	31.53 ± 5.13	0.424
Ethnicity	Malay	247 (89.5)	29 (10.5)	0.001 ^a^	167 (60.5)	109 (39.5)	0.019 ^a^	248 (89.9)	28 (10.1)	0.076 ^a^
	Non-Malay	50 (74.6)	17 (25.4)		30 (44.8)	37 (55.2)		55 (82.1)	12 (17.9)	
BMI, kg/m^2^		29.23 ± 6.30	29.12 ± 5.84	0.912	28.98 ± 5.57	29.53 ± 7.00	0.439	29.16 ± 5.96	29.59 ± 7.98	0.695
Religion	Muslim	252 (89.7)	29 (10.3)	0.000 ^a^	169 (60.1)	112 (39.9)	0.031 ^a^	253 (90.0)	28 (10.0)	0.037 ^a^
	Non-Muslim	45 (72.6)	17 (27.4)		28 (45.2)	34 (54.8)		50 (80.6)	12 (19.4)	
Education	Secondary and below	151 (84.8)	27 (15.2)	0.321	102 (57.3)	76 (42.7)	0.959	155 (87.1)	23 (12.9)	0.450
	Tertiary	146 (88.5)	19 (11.5)		95 (57.6)	70 (42.4)		148 (89.7)	17 (10.3)	
Employment	Unemployed	115 (85.8)	19 (14.2)	0.738	79 (59.0)	55 (41.0)	0.648	116 (86.6)	18 (13.4)	0.413
	Employed	182 (87.1)	27 (12.9)		118 (56.5)	91 (43.5)		187 (89.5)	22 (10.5)	
Family Income, Ringgit Malaysia		3714.90 ± 2400.77	3763.41 ± 3427.06	0.910	3638.01 ± 2490.53	3829.04 ± 2635.63	0.513	3690.32 ± 2397.41	3951.35 ± 3531.63	0.665
Pregnancy Planned	No	212 (88.7)	27 (11.3)	0.082 ^a^	142 (59.4)	97 (40.6)	0.261	214 (89.5)	25 (10.5)	0.293
	Yes	85 (81.7)	19 (18.3)		55 (52.9)	49 (47.1)		89 (85.6)	15 (14.4)	
Marital Status	Without husband	9 (64.3)	5 (35.7)	0.027 ^b^	8 (57.1)	6 (42.9)	0.982	10 (71.4)	4 (28.6)	0.067 ^b^
	With husband	288 (87.5)	41 (12.5)		189 (57.4)	140 (42.6)		450(90.0)	50(10.0)	
Parity	Nulliparous-Primiparous	161 (85.6)	27 (14.4)	0.569	100 (53.2)	88 (46.8)	0.080 ^a^	165 (87.8)	23 (12.2)	0.716
	Multiparous ≥ 2	136 (87.7)	19 (12.3)		97 (62.6)	58 (37.4)		138 (89.0)	17 (11.0)	
Smoking habit	No	291 (86.4)	46 (13.6)	1.000	191 (56.7)	146 (43.3)	0.040 ^b^	297 (88.1)	40 (11.9)	1.000
	Yes	6 (100.0)	0 (0.0)		6 (100.0)	0(0.0)		6 (100.0)	0 (0.0)	
Drink alcohol	No	291 (86.6)	45 (13.4)	1.000	193 (57.4)	143 (42.6)	1.000	297 (88.4)	39 (11.6)	0.584
	Yes	6 (85.7)	1 (13.3)		4 (57.1)	3 (42.9)		6 (85.7)	1 (14.3)	
***Past Obstetric History***										
Abortion	No	225 (88.2)	30 (11.8)	0.128 ^a^	150 (58.8)	105 (41.2)	0.376	226 (88.6)	29 (11.4)	0.776
	Yes	72 (81.8)	16 (18.2)		47 (53.4)	41 (46.6)		77 (87.5)	11 (12.5)	
Macrosomia	No	290 (86.3)	46 (13.7)	0.600	192 (57.1)	144 (42.9)	0.703	296 (88.1)	40 (11.9)	1.000
	Yes	7 (100.0)	0 (0.0)		5 (71.4)	2 (28.6)		7 (100.0)	0 (0.0)	
Gestational hypertension	No	283 (86.5)	44 (13.5)	1.000	188 (57.5)	139 (42.5)	0.922	289 (88.4)	38 (11.6)	1.000
	Yes	14 (87.5)	2 (12.5)		9 (56.3)	7 (43.8)		14 (87.5)	2 (12.5)	
Stillbirth	No	284 (86.6)	44 (13.4)	1.000	187 (57.0)	141 (43.0)	0.460	289 (88.1)	39 (11.9)	1.000
	Yes	13 (86.7)	2 (13.3)		10 (66.7)	5 (33.3)		14 (93.3)	1 (6.7)	
Preterm Delivery	No	284 (86.6)	44 (13.4)	1.000	190 (57.9)	138 (42.1)	0.388	289 (88.1)	39 (11.9)	1.000
	Yes	13 (86.7)	2 (13.3)		7 (46.7)	8 (53.3)		14 (93.3)	1 (6.7)	
Gestational Diabetes Mellitus	No	230 (87.1)	34 (12.9)	0.597	153 (58.0)	111 (42.0)	0.722	239 (90.5)	25 (9.5)	0.021 ^a^
	Yes	67 (84.8)	12 (15.2)		44 (55.7)	35 (44.3)		64 (81.0)	15 (19.0)	
***Current Medical Problems***										
Hypertension	No	284 (86.6)	44 (13.4)	1.000	188 (57.3)	140 (42.7)	0.837	291 (88.7)	37 (11.3)	0.398
	Yes	13 (86.7)	2 (13.3		9 (60.0)	6 (40.0)		12 (80.0)	3 (20.0)	
Allergy	No	294 (87.5)	42 (12.5)	0.007 ^b^	195 (58.0)	141 (42.0)	0.141 ^b^	300 (89.3)	36 (10.7)	0.004 ^b^
	Yes	3 (42.9)	4 (57.1)		2 (28.6)	5 (71.4)		3 (42.9)	4 (57.1)	
Asthma	No	273 (86.9)	41 (13.1)	0.567	186 (59.2)	128 (40.8)	0.026 ^a^	280 (89.2)	34 (10.8)	0.128 ^b^
	Yes	24 (82.8)	5 (17.2)		11 (37.9)	18 (62.1)		23 (79.3)	6 (20.7)	
Heart disease	No	291 (86.4)	46 (13.6)	1.000	192 (57.0)	145 (43.0)	0.246 ^b^	297 (88.1)	40 (11.9)	1.000
	Yes	6 (100.0)	0 (0.0)		5 (83.3)	1 (16.7)		6 (100.0)	0 (0.0)	
Anaemia	No	278 (86.6)	43 (13.4)	1.000	183 (57.0)	138 (43.0)	0.543	282 (87.9)	39 (12.1)	0.491
	Yes	19 (86.4)	3 (13.6)		14 (63.6)	8 (36.4)		21 (95.5)	1 (4.5)	
Thalassemia	No	294 (86.5)	46 (13.5)	1.000	196 (57.6)	144 (42.4)	0.577	300 (88.2)	40 (11.8)	1.000
	Yes	3 (100.0)	0 (0.0)		1 (33.3)	2 (66.7)		3 (100.0)	0 (0.0)	
***Family History***										
Diabetes mellitus	No	133 (88.1)	18 (11.9)	0.473	88 (58.3)	63 (41.7)	0.779	136 (90.1)	15 (9.9)	0.377
	Yes	164 (85.4)	28 (14.6)		109 (56.8)	83 (43.2)		167 (87.0)	25 (13.0)	
Heart Disease	No	250 (86.5)	39 (13.5)	0.916	170 (58.8)	119 (41.2)	0.229 ^a^	255 (88.2)	34 (11.8)	0.891
	Yes	47 (87.0)	7 (13.0)		27 (50.0)	27 (50.0)		48 (88.9)	6 (11.1)	
Hypertension	No	138 (85.7)	23 (14.3)	0.655	88 (54.7)	73 (45.3)	0.328	142 (88.2)	19 (11.8)	0.940
	Yes	159 (87.4)	23 (12.6)		109 (59.9)	73 (40.1)		161 (88.5)	21 (11.5)	
Depression and Anxiety	No	290 (87.6)	41 (12.4)	0.013 ^b^	193 (58.3)	138 (41.7)	0.086 ^a^	294 (88.8)	37 (11.2)	0.153 ^b^
	Yes	7 (58.3)	5 (41.7)		4 (33.3)	8 (66.7)		9 (75.0)	3 (25.0)	
Gestational Diabetes Mellitus	No	194 (88.6)	25 (11.4)	0.149 ^a^	128 (58.4)	91 (41.6)	0.614	196 (89.5)	23 (10.5)	0.374
	Yes	103 (83.1)	21 (16.9)		69 (55.6)	55 (42.6)		107 (86.3)	17 (13.7)	

Data are presented as either n (%) or mean ± SD. ^a^ Pearson Chi-Square at *p* < 0.25 entered multivariate logistic regression; ^b^ Fisher’s Exact Test at *p* < 0.25 entered multivariate logistic regression.

**Table 2:** Analyses of the *EPHX2, NPY5R, ANO2, NRG1, FKBP5, RORA, OXTR* and *BDNF* genotype among women with GDM were stratified by psychological symptoms.

Candidate Genes	SNP	Genotype	Normal	Presence of Depression Symptoms	*p*-Value	Normal	Presence of Anxiety Symptoms	*p*-Value	Normal	Presence of Stress Symptoms	*p*-Value
EPHX2	rs17466684	GG	223 (75.1)	36 (78.3)	0.122	155 (78.7)	104 (71.2)	0.267	228 (75.2)	31 (77.5)	0.078
		GA	68(22.9)	7 (15.2)		38(19.3)	37 (25.3)		69 (22.8)	6 (15.0)	
		AA	6 (2.0)	3 (6.5)		4 (2.0)	5 (3.5)		6 (2.0)	3 (7.5)	
		GG genotype	223 (75.1)	36 (78.3)	0.641	155 (78.7)	104 (71.2)	0.113	228 (75.2)	31 (77.5)	0.756
		A carrier	74 (24.9)	10 (21.7)		42 (21.3)	42 (28.8)		75 (24.8)	9 (22.5)	
		G carrier	291 (98.0)	43 (93.5)	0.106 *	193 (98.0)	141 (96.6)	0.504 *	297 (98.0)	37 (92.5)	0.075 *
		AA genotype	6 (2.0)	3 (6.5)		4 (2.0)	5 (3.4)		6 (2.0)	3 (7.5)	
NPY5R	rs12501691	TT	202 (68.0)	32 (69.5)	0.972	137 (69.6)	97 (66.4)	0.550	202 (66.7)	32 (80.0)	0.197
		TA	89 (30.0)	13 (28.3)		55 (27.9)	47 (32.2)		95 (31.3)	7 (17.5)	
		AA	6 (2.0)	1 (2.2)		5 (2.5)	2 (1.4)		6 (2.0)	1 (2.5)	
		TT genotype	202 (68.0)	32 (69.6)	0.833	137 (69.5)	97 (66.4)	0.541	202 (66.7)	32 (80.0)	0.089
		A carrier	95 (32.0)	14 (30.4)		60 (30.5)	49 (33.6)		101 (33.3)	8 (20.0)	
		T carrier	291 (98.0)	45 (97.8)	1.000	192 (97.5)	144 (98.6)	0.703 *	297 (98.0)	39 (97.5)	0.584
		AA genotype	6 (2.0)	1 (2.2)		5 (2.5)	2 (1.4)		6 (2.0)	1 (2.5)	
ANO2	rs12579350	GG	261 (87.9)	36(78.3)	0.107	168 (85.3)	129 (88.3)	0.704	263 (86.8)	34 (85.0)	0.730
		GA	33 (11.1)	10 (21.7)		27 (13.7)	16 (11.0)		37 (12.2)	6 (15.0)	
		AA	3 (1.0)	0 (0.0)		2 (1.0)	1 (0.7)		3 (1.0)	0 (0.0)	
		GG genotype	261 (87.9)	36 (78.3)	0.075	168 (85.3)	129 (88.4)	0.408	263 (86.8)	34 (85.0)	0.754
		A carrier	36 (12.1)	10 (21.7)		29 (14.7)	17 (11.6)		40 (13.2)	6 (15.0)	
		G carrier	294 (99.0)	46 (100.0)	1.000	195 (99.0)	145 (99.3)	1.000 *	300 (99.0)	40 (100.0)	1.000
		AA genotype	3 (1.0)	0 (0.0)		2 (1.0)	1 (0.7)		3 (1.0)	0 (0.0)	
NRG1	rs2919375	TT	119 (40.2)	18 (39.1)	0.812	78 (39.8)	59 (40.4)	0.981	119 (39.4)	18 (45.0)	0.097
		TC	136 (45.9)	23 (50.0)		92 (46.9)	67 (45.9)		138 (45.7)	21 (52.5)	
		CC	41 (13.9)	5 (10.9)		26 (13.3)	20 (13.7)		45 (14.9)	1 (2.5)	
		TT genotype	119 (40.2)	18 (39.1)	1.000	78 (39.8)	59 (40.4)	0.909	119 (39.4)	18 (45.0)	0.497
		C carrier	177 (59.8)	28 (60.9)		118 (60.2)	87 (59.6)		183 (60.6)	22 (55.0)	
		T carrier	255 (86.1)	41 (89.1)	0.581	170 (86.7)	126 (86.3)	0.908	257 (85.1)	39 (97.5)	0.031 *
		CC genotype	41 (13.9)	5 (10.9)		26 (13.3)	20 (13.7)		45 (14.9)	1 (2.5)	
FKBP5	rs3800373	TT	122 (41.8)	23 (50.0)	0.097	82 (42.5)	63 (43.4)	0.982	122 (40.9)	23 (57.5)	0.103
		TG	146 (50.0)	16 (34.8)		93 (48.2)	69 (47.6)		149 (50.0)	13 (32.5)	
		GG	24 (8.2)	7 (15.2)		18 (9.3)	13 (9.0)		27 (9.1)	4 (10.0)	
		TT genotype	122 (41.8)	23 (50.0)	0.295	82 (42.5)	63 (43.4)	0.86	122 (40.9)	23 (57.5)	0.047
		G carrier	170 (58.2)	23 (50.0)		111 (57.5)	82 (56.6)		176 (59.1)	17 (42.5)	
		T carrier	268 (91.8)	39 (84.8)	0.164 *	175 (90.7)	132 (91.0)	0.909	271 (90.9)	36 (90.0)	0.774
		GG genotype	24 (8.2)	7 (15.2)		18 (9.3)	13 (9.0)		27 (9.1)	4 (10.0)	
RORA	rs4775340	GG	186 (62.9)	31 (67.4)	0.775	127 (64.5)	90 (62.1)	0.818	188 (62.3)	29 (72.5)	0.449
		GA	99 (33.4)	14 (30.4)		65 (32.5)	49 (33.8)		103 (34.1)	10 (25.0)	
		AA	11 (3.7)	1 (2.2)		6 (3.0)	6 (4.1)		11 (3.6)	1 (2.5)	
		GG genotype	186 (62.8)	31 (67.4)	0.551	127 (64.5)	90 (62.1)	0.649	188 (62.3)	29 (72.5)	0.206
		A carrier	110 (37.2)	15 (32.6)		70 (35.5)	55 (37.9)		114 (37.7)	11 (27.5)	
		G carrier	285 (96.3)	45 (97.8)	1.000 *	191 (97.0)	139(95.9)	0.587	291 (96.4)	39 (97.5)	1.000 *
		AA genotype	11 (3.7)	1 (2.2)		6 (3.0)	6 (4.1)		11 (3.6)	1 (2.5)	
OXTR	rs53576	AA	76 (25.7)	16 (34.8)	0.137	49(24.9)	43(29.7)	0.611	81 (26.8)	11 (27.5)	0.337
		AG	114 (48.6)	24 (52.2)		99 (50.3)	69 (47.6)		145 (48.0)	23 (57.5)	
		GG	76 (25.7)	6 (13.0)		49 (24.9)	33 (22.8)		76 (25.2)	6 (15.0)	
		AA genotype	76 (25.7)	16 (34.8)	0.195	49 (24.9)	43 (29.7)	0.324	81 (26.8)	11 (27.5)	1.000 *
		G carrier	220 (74.3)	30 (65.2)		148 (75.1)	102 (70.3)		221 (73.2)	29 (72.5)	
		A carrier	220 (74.3)	40 (87.0)	0.062	148 (75.1)	112 (77.2)	0.651	226 (74.8)	34 (85.0)	0.157
		GG genotype	76 (25.7)	6 (13.0)		49 (24.9)	33 (22.8)		76 (25.2)	6 (15.0)	
BDNF	rs6265	GG	95 (32.1)	19 (42.2)	0.361	62 (31.4)	52 (36.1)	0.646	96 (31.9)	18 (45.0)	0.230
		GA	145 (49.0)	20 (44.4)		99 (50.3)	66 (45.8)		148 (49.2)	17 (42.5)	
		AA	56 (18.9)	6 (13.3)		36 (18.3)	26 (18.1)		57 (18.9)	5 (12.5)	
		GG genotype	95 (32.1)	19 (42.2)	0.180	62 (31.5)	52 (36.1)	0.370	96 (31.9)	18 (45.0)	0.099
		A carrier	201 (67.9)	26 (57.8)		135 (68.5)	92 (63.9)		205 (68.1)	22 (55.0)	
		G carrier	240 (81.1)	39 (86.7)	0.365	161 (81.7)	118 (81.9)	0.959	244 (81.1)	35 (87.5)	0.321
		AA genotype	56 (18.9)	6 (13.3)		36 (18.3)	26 (18.1)		57 (18.9)	5 (12.5)	
FKBP5	rs9470080	CC	128 (43.0)	22 (47.8)	0.681	85 (42.9)	65 (44.5)	0.953	127 (41.8)	23 (57.5)	0.160
		CT	137 (46.0)	18 (39.2)		90 (45.5)	65 (44.5)		142 (46.7)	13 (32.5)	
		TT	33 (11.0)	6 (13.0)		23 (11.6)	16 (11.0)		35 (11.5)	4 (10.0)	
		CC genotype	128 (43.0)	22 (47.8)	0.535	85 (42.9)	65(44.5)	0.769	127 (41.8)	23 (57.5)	0.059
		T carrier	170 (57.0)	24 (52.2)		113 (57.1)	81 (55.5)		177 (58.2)	17 (42.5)	
		C carrier	265 (88.9)	40 (87.0)	0.695	175 (88.4)	130 (89.0)	0.849	269 (88.5)	36 (90.0)	1.000 *
		TT genotype	33 (11.1)	6 (13.0)		23 (11.6)	16 (11.0)		35 (11.5)	4 (10.0)	

Note: * *p-*value based on fisher’s exact test.

**Table 3:** Multiple regression analysis between genotypes of candidate genes and the presence of psychological symptoms adjusted for the confounding factors (n = 343).

Candidate Genes SNP	Geno-Types	Depression Symptoms		Geno-Types	Anxiety Symptoms		Geno-Types	Stress Symptoms	
		Crude OR (95% CI), *p-*Value	Adjusted OR (95% CI), *p-*Value		Crude OR (95% CI), *p-*Value	Adjusted OR (95% CI), *p-*Value		Crude OR (95% CI), *p-*Value	Adjusted OR (95% CI), *p-*Value
*EPHX2* *rs17466684*	GG/GA	1	1	GG	1	1	GG/GA	1	1
	AA	3.846 (0.852–17.353), 0.080	7.854 (1.330–46.360), 0.023	AA/AG	1.490 (0.909–2.444), 0.114	1.580 (0.943–2.659), 0.083	AA	4.622 (0.964–22.158), 0.056	7.664 (1.579–37.197), 0.012
*ANO2* *rs12579350*	GG	1	1	-	-	-	-	-	-
	AA/AG	2.037 (0.907–4.573), 0.085	1.880 (0.655–5.393), 0.240	-	-	-	-	-	-
*FKBP5* *rs3800373*	TT/TG	1	1	-	-	-	GG/GT	1	1
	GG	1.879 (0.729–4.841), 0.192	2.497 (0.746–8.359), 0.138	-	-	-	TT	1.446 (0.255–8.193), 0.677	1.963 (0.952–4.045), 0.068
*OXTR* *rs53576*	GG	1	1	-	-	-	GG	1	1
	AA/AG	2.490 (0.988–6.274), 0.053	2.114 (0.704–6.348), 0.182	-	-	-	AA/AG	2.228 (0.8595–5.779), 0.099	2.981 (1.058–8.402), 0.039
*BDNF* *rs6265*	AA/AG	1	1	-	-	-	AA/AG	1	1
	GG	1.498 (0.778–2.885), 0.227	1.045 (0.429–2.548), 0.922	-	-	-	GG	1.883 (0.932–3.802), 0.078	1.651 (0.786–3.468), 0.185
*NPY5R* *rs12501691*	-	-	-	-	-	-	AA/AT	1	1.000
	-	-	-	-	-	-	TT	2.206 (0.948–5.136),0.066	2.182 (0.915–5.204), 0.079
*NRG1* *rs2919375*	-	-	-	-	-	-	CC	1	1
	-	-	-	-	-	-	TT/TC	7.752 (1.000–60.105), 0.050	9.894 (1.159–84.427), 0.036
*FKBP5 rs9470080*	-	-	-	-	-	-	TT/TC	1	1
	-	-	-	-	-	-	CC	1.539 (0.271–8.739), 0.627	1.118 (0.161–7.762), 0.910
*RORA* *rs4775340*	-	-	-	-	-	-	AA/AG	1	1
	-	-	-	-	-	-	GG	1.822 (0.848–3.914), 0.124	1.790 (0.789–4.061), 0.164

Note: Adjusted OR was determined by adjusting for socio-demographical and clinical moderators with *p-*value < 0.25 in univariate analysis.

## Appendix A

**Table A1:** Candidate Genes and Single Nucleotide Polymorphism (SNP) Details.

Candidate Genes	SNP	Chromosome: Location	Sequence of SNP (60 upstream, 60 downstream)
Epoxide Hydrolase 2	rs17466684	8:27595330	CCGTGGAGAC CCAAGTCCTC TTTGCATTGT CTCTAGAACT ACTGGATACT TCCTGGGTTT
			A/G
			CCACTATCCT ATTTTCTAGT GGGGCCCTGT GATCCCCAGA GACAGACCCG TGTTCATTCT
Neuropeptide Y	rs12501691	4:163346876	GTAAATATAT CTTACAGTTT TAGTTGCATG TTGCTTGTGT GATAGCCTTT ATCAATGAAG
			A/T
			TATCCAAATT TAAAGTGCTA AACTATCTTT ATTGTCTGTC TAGGTATCTC CTCCTCATTG
Anoctamin 2	rs12579350	12:5687935	AACAACACCA GGAGGTCAGG TCCAATGTCC CACACTGGTT CCCTCTCCTG ACTTTGCCTT
			A/G
			ACCTTGTGTT GAGATTTAAA AGCATTAAAG AAAGGTATAT ATTATAAGGA CTGCTGAATT
Neuregulin 1	rs2919375	8:32719327	AAACAAAACT GATAACGGCT GAAGTGGGTG ATGGCTACAT GGAGATTCAT TACACAATCC
			C/T
			TTGTATTTTC AGGTTTTTAA TATGCATGTT TAAATGGATA TTATATATGT ACTTGTTTAA
FK506 binding protein 5	rs3800373	6: 35574699	CATGCAAAAA AATTTTGACT TTTTAGTACT AAGCTTAATT TTTAAAAACA AAATCTGTAG
			G/T
			GTTGACAAAT AAATAGTTGC TCTTCTACAC TAGGGGTTTC ACCTGCAGGT TTGACACGCA
retinoid-related orphan receptor alpha	rs4775340	15:60975553	AAACAGTAAG AAAATTGGAT CCTAGAACTC ACTCTGGAGA ACACTGAAAT GAACATGTGG
			A/G
			GTCCTATTCA GAACATGTTT GCCTTGAGTG TATGGAATCT GGGTCACCTT CACTGAAAGC
oxytocin receptor genes	rs53576	3:8762685	TCCCCCACAC CTCGGGCACA GCATTCATGG AAAGGAAAGG TGTACGGGAC ATGCCCGAGG
			A/G
			TCCTCAGTCC CACAGAAACA GGGAGGGGCT GGGAAGCTCA TTCTACAGAT GGGGAAACAG
Brain-derived neurotrophic factor	rs6265	11:27658369	GTGAATGGGC CCAAGGCAGG TTCAAGAGGC TTGACATCAT TGGCTGACAC TTTCGAACAC
			A/G
			TGATAGAAGA GCTGTTGGAT GAGGACCAGA AAGTTCGGCC CAATGAAGAA AACAATAAGG
FK506 binding protein 5	rs9470080	6: 35678658	ATTGACAAAA AGCAGCTAAA GACAAAAACA GTTTCATAAT TACCATTTGT CCAAAGTCAA
			C/T
			CTCTGAGCTA AAACACAATG TTTTTTATGT TTCTCTACTT ATAACAAAAT TTCGGGAAAA
Tryptophan hydroxylase 2	rs1843809	12:71954918	TAGTTATTTC AATCCATCTT ATTCTCTTGG AAAGAGGCCC TGAGCTCCTA CTTTAATTAT
			G/T
			CCACTCTTGT TTGCTTAAAT TGATTTTGAA TATTATTGTG ATTGTGTTTT ATTATGAATG
Catenin Alpha 3	rs10997242	10:66576537	CCCACCACCC TCCCCAATGA AGCAGTCTCC AGAGTCTTTG TTCCTATCTT TGTGTCCATT
			C/T
			ATATTCAATG TTGAGCTTCC AATTATAAGC GAAAACATGT GGAATGTGGT TGTCTGTTCC
Phospholysine Phosphohistidine Inorganic Pyrophosphate Phosphatase	rs35936514	10:124556401	CACCGTGCAT TCTCCGGGGC CATCGTTTTA ATGGCTGCAC CCTGCTCCCG CGTGTGGACG
			C/T
			ATCCTAAACA GTCCCTTAGT ATTATGGTTA GATGCTCCAT GTGTTTCCAA TTCTTCATTA
Calcium Voltage-Gated Channel Subunit Alpha1 C	rs1006737	12:2236129	ACTTGGCTC TATCAAAGTC TTGCTATCAA TTACATAAGT TCCATTCCAT CTCAGCCCGAA
			A/G
			TGTTTTCAGA GCCGGAGACC TCACAGTGTC TCTCAGGACA GTACCTTTCA GGTTTGAATG
Apolipoprotein L3	rs132617	22:36137737	AGCAGATAAG GAGAGTTCTT TTTGTTTGTA TGAGAAGAAG AGTGTGTGTG CAGTAGCAAG
			C/T
			GATTGACTGT ATACAATGAG CACAAATTCA GGTGGCTGTT TGGCCAGAGG CTTCCCATTA
Testis Expressed 51	rs6733840	2:126902405	GTGTGATGCT TTGGCCAGGC TGGTGTGCTC CGACCCAGGA ACCTGCCCAC CTCATATTTA
			C/T
			TGTCCAGTAT TTGGCCATGC CATGGGTGCA GATCCAAAGC CCTCACTCCC CTTTTCTCCT
Pleckstrin Homology And RhoGEF Domain Containing G1	rs9372078	6:150592825	AAGCAGCTGG GGTGGACTTA CAGGAACTGG ACACAAGTCC CTGATTTGGA GTGTTTGCCA
			A/T
			TTTTTGTGGT GTAAATATCT CCACCATGGC TGATTTCAAG CCACCAATGT GATGTCAGTT
5-Hydroxytrytamine receptor 2	rs6318	X: 114731326	GATTGTTTTT TTTTTTCTTA ATTTTCAGTG TGCACCTAAT TGGCCTATTG GTTTGGCAAT
			C/G
			TGATATTTCT GTGAGCCCAG TAGCAGCTAT AGTAACTGAC ATTTTCAATA CCTCCGATGG
Sidekick Cell Adhesion Molecule 2	rs3816995	17:73339121	ACTGTGGGCC TCCCAGCCCC CTCACTGCCA AGGGGGTCTG GTGCCCGTTT GTGCCCGCCT
			A/G
			CTGCTTCCTT CACAGCAGAT CCGGAACCGG AAGGATCTAC TATGGGGTTG GCCCAGAGCT

**Table A2:** Genotype and allelic information for candidate genes and its chi-squared goodness-of-fit based global distribution (n = 343).

Candidate Genes	SNP	Genotype	Expected Genotype Frequency	Expected N	Frequency	N	Allele	Frequency	Call Rate, %	*p*-Value Chi-Squared Goodness-of-Fit
*BDNF*	rs6265	GG	0.466	160	0.334	114	G A	0.576 0.424	98.9	0.69
		GA	0.333	114	0.484	165				
		AA	0.201	69	0.182	62				
*OXTR*	rs53576	AA	0.389	134	0.269	92	A G	0.515 0.485	98.9	0.68
		AG	0.333	114	0.491	168				
		GG	0.278	95	0.240	82				
*RORA*	rs4775340	GG	0.450	155	0.635	217	G A	0.800 0.200	99.2	0.43
		GA	0.333	114	0.330	113				
		AA	0.217	74	0.035	12				
*NRG1*	rs2919375	TT	0.388	133	0.401	137	T C	0.634 0.366	99.2	0.86
		TC	0.333	114	0.465	159				
		CC	0.279	96	0.135	46				
*TPH2*	rs1843809	TT	0.514	177	0.915	312	T G	0.958 0.042	99.2	0.43
		GT	0.333	114	0.085	29				
		GG	0.153	52	0.000	0				
*LHPP*	rs35936514	CC	0.593	204	0.474	162	C T	0.683 0.317	99.2	0.45
		CT	0.333	114	0.418	143				
		TT	0.074	25	0.108	37				
*FBKP5*	rs9470080	CC	0.363	125	0.436	150	C T	0.662 0.338	100	0.73
		CT	0.333	114	0.451	155				
		TT	0.304	104	0.113	39				
*FBKP5*	rs3800373	TT	0.425	146	0.429	145	T G	0.669 0.331	98.4	0.17
		TG	0.333	114	0.479	162				
		GG	0.242	83	0.092	31				
*TEX51*	rs6733840	TT	0.488	168	0.638	219	C T	0.796 0.204	99.7	0.68
		TC	0.333	114	0.315	108				
		CC	0.178	61	0.047	16				
*PLEKHGI*	rs9372078	AA	0.384	131	0.388	132	A T	0.624 0.376	98.4	0.78
		AT	0.333	114	0.471	160				
		TT	0.283	97	0.141	48				
*HTR2C*	rs6318	GG	0.571	196	0.944	323	G C	0.971 0.029	99.5	0.63
		GC	0.333	114	0.053	18				
		CC	0.095	33	0.003	1				
*EPHX2*	rs17466684	GG	0.536	184	0.755	259	G A	0.864 0.136	99.7	0.19
		GA	0.333	114	0.219	75				
		AA	0.131	45	0.026	9				
*ANO2*	rs12579350	GG	0.541	186	0.860	297	G A	0.923 0.077	100.0	0.28
		GA	0.333	114	0.125	43				
		AA	0.126	43	0.009	3				
*NPY5R*	rs12501691	TT	0.613	210	0.682	234	T A	0.831 0.169	99.5	0.18
		TA	0.333	114	0.297	102				
		AA	0.054	19	0.020	7				
*SDK2*	rs3816995	GG	0.406	140	0.617	211	G A	0.779 0.221	99.2	0.39
		GA	0.333	114	0.325	111				
		AA	0.260	89	0.058	20				

**Table A3:** Analyses of the genotype of LHPP, SDK2, HTR2C, TEX51, PLEKHG1 and TPH2 among women with GDM were stratified by presence of psychological symptoms. * *p*-value based on fisher’s exact test.

Candidate Genes	SNP	Genotype	Normal	Presence of Depression Symptoms	*p*-Value	Normal	Presence of Anxiety Symptoms	*p*-Value	Normal	Presence of Stress Symptoms	*p*-Value
*LHPP*	rs35936514	CC	139 (85.8)	23 (14.2)	0.600	97 (59.9)	65 (40.1)	0.262	144 (88.9)	18 (11.1)	0.909
		CT	123 (86.0)	20 (14.0)		75 (52.4)	68 (47.6)		125 (87.4)	18 (12.6)	
		TT	34 (91.9)	3 (8.1)		24 (64.9)	13 (35.1)		33 (89.2)	4 (10.8)	
		CC genotype	139 (85.8)	23 (14.2)	0.701	97 (59.9)	65 (40.1)	0.363	144 (88.9)	18 (11.1)	0.750
		T carrier	157 (87.2)	23 (12.8)		99 (55.0)	81 (45.0)		158 (87.8)	22 (12.2)	
		C carrier	262 (85.9)	43 (14.1)	0.445 *	172(56.4)	133 (43.6)	0.325	269 (88.2)	36 (11.8)	1.000 *
		TT genotype	34 (91.9)	3 (8.1)		24 (65.9)	13 (35.1)		33 (89.2)	4 (10.8)	
*SDK2*	rs3816995	GG	183 (86.7)	28 (13.3)	0.910	119 (56.4)	92 (43.6)	0.735	187 (88.6)	24 (11.4)	0.920
		GA	96 (86.5)	15 (13.5)		65 (58.6)	46 (41.4)		97 (87.4)	14 (12.6)	
		AA	18 (90.0)	2 (10.0)		13 (65.0)	7 (35.0)		18 (90.0)	2 (10.0)	
		GG genotype	183 (86.7)	28 (13.3)	0.938	119 (56.4)	92 (43.6)	0.567	187 (88.6)	24 (11.4)	0.814
		A carrier	114 (87.0)	17 (13.0)		78 (59.5)	53 (40.5)		115 (87.8)	16 (12.2)	
		G carrier	279 (86.6)	43 (13.4)	1.000 *	184 (57.1)	138 (42.9)	0.490	284 (88.2)	38 (11.8)	1.000 *
		AA genotype	18 (90.0)	2 (10.0)		13 (65.0)	7 (35.0)		18 (90.0)	2 (10.0)	
*HTR2C*	rs6318	GG	279 (86.4)	44 (13.6)	0.883	187 (57.9)	136 (42.1)	0.496 *	286 (88.5)	37 (11.5)	0.748
		GC	16 (88.9)	2 (11.1)		10 (55.6)	8 (44.4)		15 (83.3)	3 (16.7)	
		CC	1 (100.0)	0 (0.0)		0 (0.0)	1 (100.0)		1 (100.0)	0 (0.0)	
		GG genotype	279 (86.4)	44 (13.6)	1.000 *	187 (57.9)	136 (42.1)	0.652	286 (88.5)	37 (11.5)	0.475 *
		C carrier	17 (89.5)	2 (10.5)		10 (52.6)	9 (47.4)		16 (84.2)	3 (15.8)	
		G carrier	295 (86.5)	46 (13.5)	1.000 *	197 (57.8)	144 (42.2)	0.424 *	301 (88.3)	40 (11.7)	1.000 *
		CC genotype	1 (100.0)	0 (0.0)		0 (0.0)	1 (100.0)		1(100.0)	0 (0.0)	
*TEX51*	rs6733840	TT	189 (86.3)	30 (13.7)	0.977	125 (57.1)	94 (42.9)	0.914	191 (87.2)	28 (12.8)	0.643
		TC	94 (87.0)	14 (13.0)		62 (57.4)	46 (42.6)		98 (90.7)	10 (9.3)	
		CC	14 (87.5)	2 (12.5)		10 (62.5)	6 (37.5)		14 (87.5)	2 (12.5)	
		TT genotype	189 (86.3)	30 (13.7)	0.835	125 (57.1)	94 (42.9)	0.859	191 (87.2)	28 (12.8)	0.389
		C carrier	108 (87.1)	16 (12.9)		72 (58.1)	52 (41.9)		112 (90.3)	12 (9.7)	
		T carrier	282 (86.5)	44 (13.5)	1.000 *	187 (57.2)	140 (42.8)	0.675	289 (88.4)	38 (11.6)	1.000 *
		CC genotype	14 (87.5)	2 (12.5)		10 (62.5)	6 (37.5)		14 (87.5)	2 (12.5)	
*PLEKHG1*	rs9372078	AA	115 (87.1)	17 (12.9)	0.951	75 (56.8)	57 (43.2)	0.878	118 (89.4)	14 (10.6)	0.763
		AT	138 (86.3)	22 (13.8)		95 (59.4)	65 (40.6)		139 (86.9)	21 (13.1)	
		TT	41 (85.4)	7 (14.6)		27 (56.3)	21 (43.8)		43 (89.6)	5 (10.4)	
		AA genotype	115 (87.1)	17 (12.9)	0.780	75 (56.8)	57 (43.2)	0.738	118 (89.4)	14 (10.6)	0.597
		T carrier	179 (86.1)	29 (13.9)		122 (58.7)	86 (41.3)		182 (87.5)	26 (12.5)	
		A carrier	253 (86.6)	39 (13.4)	0.818	170 (58.2)	122 (41.8)	0.798	257 (88.0)	35 (12.0)	0.754
		TT genotype	41 (85.4)	7 (14.6)		27 (56.3)	21 (43.8)		43 (89.6)	5 (10.4)	
*TPH2*	rs1843809	TT	269 (86.2)	43 (13.8)	0.398 *	179(57.4)	133 (42.6)	0.896	274 (87.8)	38 (12.2)	0.553 *
		TG	27 (93.1)	2 (6.9)		17 (58.6)	12 (41.4)		27 (93.1)	2 (6.9)	
		GG	0 (0.0)	0 (0.0)		0 (0.0)	0 (0.0)		0 (0.0)	0 (0.0)	
		TT genotype	-	-		-	-		-	-	
		G carrier	-	-		-	-		-	-	
		T carrier	-	-		-	-		-	-	
		GG genotype	-	-		-	-		-	-	
